# LINC01106 drives colorectal cancer growth and stemness through a positive feedback loop to regulate the Gli family factors

**DOI:** 10.1038/s41419-020-03026-3

**Published:** 2020-10-16

**Authors:** Kun Guo, Wenbin Gong, Qin Wang, Guosheng Gu, Tao Zheng, Ying Li, Weijie Li, Miao Fang, Haohao Xie, Chao Yue, Jianbo Yang, Zhiqiang Zhu

**Affiliations:** 1 Department of General Surgery, the First Affiliated Hospital of USTC, Division of Life Sciences and Medicine, University of Science and Technology of China, 230001 Hefei, Anhui Province P. R. China; 2 Department of General Surgery, Jinling Hospital, School of Medicine, Southeast University, 210009 Nanjing, Jiangsu Province P. R. China; 3 Institute of Clinical Physiology, Jiangsu Health Vocational College, 211800 Nanjing, Jiangsu Province P. R. China; 4 Department of General Surgery, Jinling Hospital, Nanjing Medical University, 210002 Nanjing, Jiangsu Province P. R. China; 5 Institute of Clinical Pharmacology, Anhui Medical University, 230032 Hefei, Anhui Province P. R. China; 6 Department of General Surgery, Jiangsu Cancer Hospital & Jiangsu Institute of Cancer Research & The Affiliated Cancer Hospital of Nanjing Medical University, 210009 Nanjing, Jiangsu Province P. R. China

**Keywords:** Cancer, Cell biology

## Abstract

Long non-coding RNAs (lncRNAs) are essential contributors to the progression of various human cancers. Long intergenic non-protein coding RNA 1106 is a member of lncRNAs family. Until now, the specific role of LINC01106 in CRC remains undefined. The aim the current study was to unveil the functions of LINC01106 and explore its potential molecular mechanism in CRC. Based on the data of online database GEPIA, we determined that LINC01106 was expressed at a high level in colon adenocarcinoma (COAD) tissues compared to normal colon tissues. More importantly, high level of LINC01106 had negative correlation with the overall survival of COAD patients. Additionally, we also determined the low level of LINC01106 in normal colon tissues based on UCSC database. Through qRT-PCR, we identified that LINC01106 was highly expressed in CRC tissues compared to adjacent normal ones. Similarly, we detected the expression of LINC01106 and confirmed that LINC01106 was expressed higher in CRC cells than that in normal cells. Subsequently, LINC01106 was mainly distributed in the cytoplasm. LINC01106 induced the proliferation, migration, and stem-like phenotype of CRC cells. Mechanistically, cytoplasmic LINC01106 positively modulated Gli4 in CRC cells by serving as a miR-449b-5p sponge. Furthermore, nuclear LINC01106 could activate the transcription of Gli1 and Gli2 through recruiting FUS to Gli1 and Gli2 promoters. Mechanism of investigation unveiled that Gli2 was a transcription activator of LINC01106. In conclusion, Gli2-induced upregulation of LINC01106 aggravates CRC progression through upregulating Gli2, Gli2, and Gli4.

## Introduction

Colorectal cancer (CRC) results in 600,000 fatalities each year worldwide. It ranks third among all usually diagnosed human cancers^1,2^. Due to the improvement of anti-tumor therapeutics, annual fatalities are gradually decreasing in recent decades^3^. Genetic and epigenetic alterations are recognized as factors closely associated with CRC deterioration^4,5^. Furthermore, it is predicted that the mortality of CRC will be climbing until 2035^6^. Increasing comprehension on the molecular mechanism underneath CRC progression will help to find novel methods for the treatment.

Long non-coding RNAs (lncRNAs) are RNA molecules without protein-coding capacity. Dysregulation of lncRNAs is illuminated to be associated with the biological functions, including cell growth, mobility, and stemness. For examples, lncRNA UICLM induces CRC metastasis via increasing ZEB2 expression^7^. LncRNA AB073614 accelerates in CRC cell proliferation and invasiveness^8^. LncRNA BCAR4 maintains stemness and facilitates tumorigenesis in CRC via targeting to miR-665/STAT3 signaling^9^. MicroRNAs (miRNAs) are small ncRNAs, which function in human cancers through post-transcriptionally modulating gene expression^10^. Studies suggest miR-495 suppresses CRC cell proliferation and migration by targeting FAM83D^11^. miR-144 plays essential role in suppressing CRC cell proliferation and migration through modulation of GSPT1^12^. Mechanistically, cytoplasmic lncRNAs are capable of arresting miRNA function via sequestering miRNAs to upregulate mRNAs^13^. Moreover, nuclear lncRNAs have been proven to be the scaffold for proteins to regulate downstream targets and thus affect tumor cell growth, stem-like potency, and mobility^14–16^. Based on the data of online databases, LINC01106 was dysregulated in CRC samples and associated with patients’ poor prognosis. LINC01106 has been reported to be participant in lncRNA–miRNA–mRNA axis^17^. However, the specific functions of LINC01106 in CRC progression and its upstream or downstream molecular mechanism remain unclear. Thus, the aim of this study was to unmask the mechanism by which LINC01106 functions in CRC progression.

Mechanistically, lncRNAs can regulate the expression of their downstream genes at transcriptional or post-transcriptional level. LncRNAs can act as positive regulators for mRNAs by acting as sponges for miRNAs^18–21^. In the current study, the possible downstream miRNAs of cytoplasmic LINC01106 were also detected. In addition, nuclear lncRNAs can act as scaffold and recruit transcriptional factors to the promoter region of their downstream targets^22^. Here, we also detected whether nuclear LINC01106 exert functions in CRC in the same way.

Transcriptional regulation is an important factor leading to the upregulation of lncRNAs^23–25^. In our current study, we also investigated the mechanism which induced the upregulation of LINC01106 in CRC.

To sum up, our work was aimed at unveiling the association of LINC01106 with CRC progression.

## Materials and methods

### Tissue collection

CRC tissues (*n* = 68) and adjacent non-tumor tissues (*n* = 68) were collected via surgical operation from the First Affiliated Hospital of USTC. All patients did not receive any therapy before surgery. The participants were blinded to the group allocation during the experiment. Fresh samples were snap frozen in the liquid nitrogen and preserved at −80 °C. Informed consents were signed by each participant, with the approval obtained from the Ethics Committee of the First Affiliated Hospital of USTC (Registration number: 2018NZGKJ-016).

### Cell culture and treatment

Normal colon epithelial cell line (FHC) and CRC cell lines (HT29, HCT116, SW480, LoVo and SW1116) were purchased from American Type Culture Collection (ATCC; Manassas, VA, USA). Cells were cultured in RPMI-1640 (Gibco, Grand Island, NY, USA) with 10% of fetal bovine serum (FBS; Gibco) at 37 °C in 5% CO_2_.

### Cell transfection

Specific shRNAs against LINC01106 (sh/LINC01106#1/2), FUS (sh/FUS#1/2), Gli1 (sh/Gli1#1/2), Gli2 (sh/Gli2#1/2), and their relative negative control (sh/ctrl), were obtained from Genechem (Shanghai, China), together with pcDNA3.1/Gli4, pcDNA3.1/FUS, pcDNA3.1/Gli1, pcDNA3.1/Gli2, and the pcDNA3.1 empty vector (negative control). MiRNA mimics and NC mimics, miR-449b-5p inhibitor and NC inhibitor were all synthesized by GenePharma (Shanghai, China). All above plasmids were constructed into the pLKO.1 vector (Addgene #10878), followed by lentiviral particle preparation as previously described^26^. Thereafter, lentiviral particles containing above plasmids were separately transfected into LoVo or SW1116 cells for 48 h by Lipofectamine 3000 (Invitrogen, Carlsbad, CA, USA). Finally, stably transfected cells were screened out via 2 μg/ml of puromycin treatment for additional 48 h.

### qRT-PCR

Utilizing TRIzol (Invitrogen), total RNA was isolated from LoVo and SW1116 cells and then reverse-transcribed into cDNA. qRT-PCR was conducted by using SYBR-green IMaster Mix (Roche, Mannheim, Germany). Gene expression was calculated using 2^−ΔΔCt^ and quantified by normalizing to GAPDH or U6.

### Northern blot

Northern blot assay was conducted in accordance with a previous protocol^27^.

### Fluorescence in situ hybridization (FISH)

LoVo or SW1116 cells were loaded on glass coverslips, followed by being washed with PBS (Sigma-Aldrich, St. Louis, MO, USA). Fixation for 30 min in 4% formaldehyde (Sigma-Aldrich) was later conducted. After being permeabilized using 70% ethanol (Sigma-Aldrich) for one night, cells were rinsed twice by PBS. Cells were incubated with LINC01106 probe (GenePharma) at 37 °C overnight. Cells were dyed for 10 min with DAPI (Sigma-Aldrich), washed in saline-sodium citrate (SSC; Sigma-Aldrich) and photographed by fluorescence microscope (Olympus, Tokyo, Japan).

### Subcellular fractionation

The cytoplasmic and nuclear RNA purification kit acquired from Norgen (Thorold, ON, Canada) was applied to isolate the nuclear and cytoplasmic fractions. Expression levels of GAPDH, U6, or LINC01106 in nuclear and cytoplasmic fractions of LoVo or SW1116 cells were measured using qRT-PCR.

### Colony formation assay

LoVo or SW1116 cells were plated in six-well plates (1 × 10^5^ cells/well). After the plates were incubated for 2 weeks, cells were fixed and stained with 0.1% crystal violet solution (Sigma-Aldrich). Colonies with more than 50 cells were counted manually.

### EdU assay

LoVo or SW1116 cells were seeded into 96-well plates (8000 cells/well), and 50 μM EdU reagent (RiboBio, Guangdong, China) was added for 2 h. Cells were fixed and washed with PBS before being stained for 30 min by 100 μL of Apollo (RiboBio). Cells were incubated with DAPI and assessed under the fluorescent microscope.

### Transwell assay

LoVo or SW1116 cells were suspended in serum-free medium and added into the upper chambers of 8-µm-pore Transwells (Corning, Cambridge, MA, USA). 10% FBS-medium was added into the lower chambers. Twenty-four hours later, non-migratory cells were removed using a cotton swab, whereas migratory cells were immobilized and stained. Finally, migratory cells were visualized adopting the GX41 light microscope (Olympus).

### Western blot

LoVo or SW1116 cells were lysed on ice with the lysis buffer (Beyotime, Shanghai, China) and the lysates were later centrifuged for 15 min at 12,000 × *g*. The BCA kit (Thermo Fisher Scientific, Waltham, MA, USA) was acquired for evaluating protein concentrations. Protein was subjected to SDS–PAGE (Bio-Rad, Hercules, CA, USA) and transferred to PVDF membranes (Millipore, Billerica, MA, USA). Primary antibodies and secondary antibodies were applied to incubate membranes in sequence. ECL detection system (Applied Biosystems, Foster City, CA, USA) was utilized to visualize protein bands. Primary antibodies against MMP2 (ab97779, Abcam, Cambridge, MA, USA), MMP7 (ab5706, Abcam), N-cadherin (ab76057, Abcam), E-cadherin (ab40772, Abcam), NANOG (ab80892, Abcam), OCT4 (ab181557, Abcam), Gli4 (AV37797, Sigma-Aldrich), PTCH1 (ab53715, Abcam), Shh (ab53281, Abcam), Gli1 (ab49314, Abcam), Gli2 (ab167389, Abcam), and GAPDH (ab9484, Abcam) were used, individually.

### Sphere formation assay

LoVo or SW1116 cells were cultured in 96-well ultralow attachment plates from Corning (10 cells/well) in sphere medium. Seven days later, the cell clusters with a diameter >50 mm were defined as sphere cells and counted. Images of spheres were taken by an inverted light microscope (Olympus).

### Luciferase reporter assay

The wild-type (WT) or mutant (MUT) interacting sequences of miR-449b-5p in LINC01106 or Gli4 3′-UTR were sub-cloned into pmirGLO vector (Promega, Madison, WI, USA) and co-transfected with miR-449b-5p mimics or NC mimics. The pGL3-Gli1 promoter, pGL3-Gli2 promoter or pGL3-LINC01106 promoter was constructed using pGL3-reporter vector (Promega), and co-transfected into cells with indicated transfection plasmids. Luciferase activities of all above reporter vectors were evaluated by dual luciferase reporter assay system (Promega), with Renilla luciferase as normalization.

### RNA immunoprecipitation (RIP)

RIP was conducted in LoVo or SW1116 cells in line with the instruction of RNA-binding protein immunoprecipitation kit (Millipore). Cell lysates were obtained in RIP lysis buffer and incubated with indicated antibodies. Anti-IgG antibody (Millipore) acted as the negative control. The relative RNA enrichment was estimated by qRT-PCR.

### RNA pull-down

Cell lysates obtained from LoVo or SW1116 cells were incubated with Bio-LINC01106 sense/antisense and streptavidin agarose magnetic beads (Invitrogen) at 4 °C for 1 h. After elution, the binding proteins were subjected to SDS–PAGE separation and visualized by silver staining, followed by mass spectrometry (MS) analysis and western blotting.

### MS analysis

MS analysis was performed to identify LINC01106 interactors obtained from RNA pull down assays according to the previous protocol^16^. Briefly, the bands with predominant concentration in the LINC01106 pull-down compounds were selected to be subjected to MS analysis. Then, proteins with at least two peptides sequenced were thought to be a reliable identification.

### Chromatin immunoprecipitation (ChIP)

ChIP was conducted by using the SimpleChip plus Enzymatic Chromatin IP kit (Cell Signaling Technology, Beverly, MA, USA). LoVo or SW1116 cells were cross-linked using 1.5% formaldehyde. The sheared chromatin was immunoprecipitated using anti-FUS, anti-Gli2 or anti-IgG (negative control) at 4 °C overnight. The recovered DNA upon purification was subjected to qRT-PCR.

### Immunofluorescence (IF)

LoVo or SW1116 cells were rinsed twice with PBS and fixed for 20 min in 4% paraformaldehyde (PFA; Sigma-Aldrich). Thereafter, cells were treated with anti-E-cadherin or anti-N-cadherin primary antibody, and then incubated for 1 h with secondary antibody. DAPI was adopted for nucleus staining. Finally, images were captured under a microscope (Olympus).

### Statistical analysis

Values were shown as means ± SD. Statistical significances in groups were identified with Student’s *t*-test or one-way ANOVA. *P* < 0.05 was considered statistically significant. Assays were undertaken in triplicate. Statistical analysis was performed using Prism 5 (GraphPad, La Jolla, CA, USA).

## Results

### Highly expressed LINC01106 is correlated with the poor prognosis of CRC patients

**Fig. 1:**
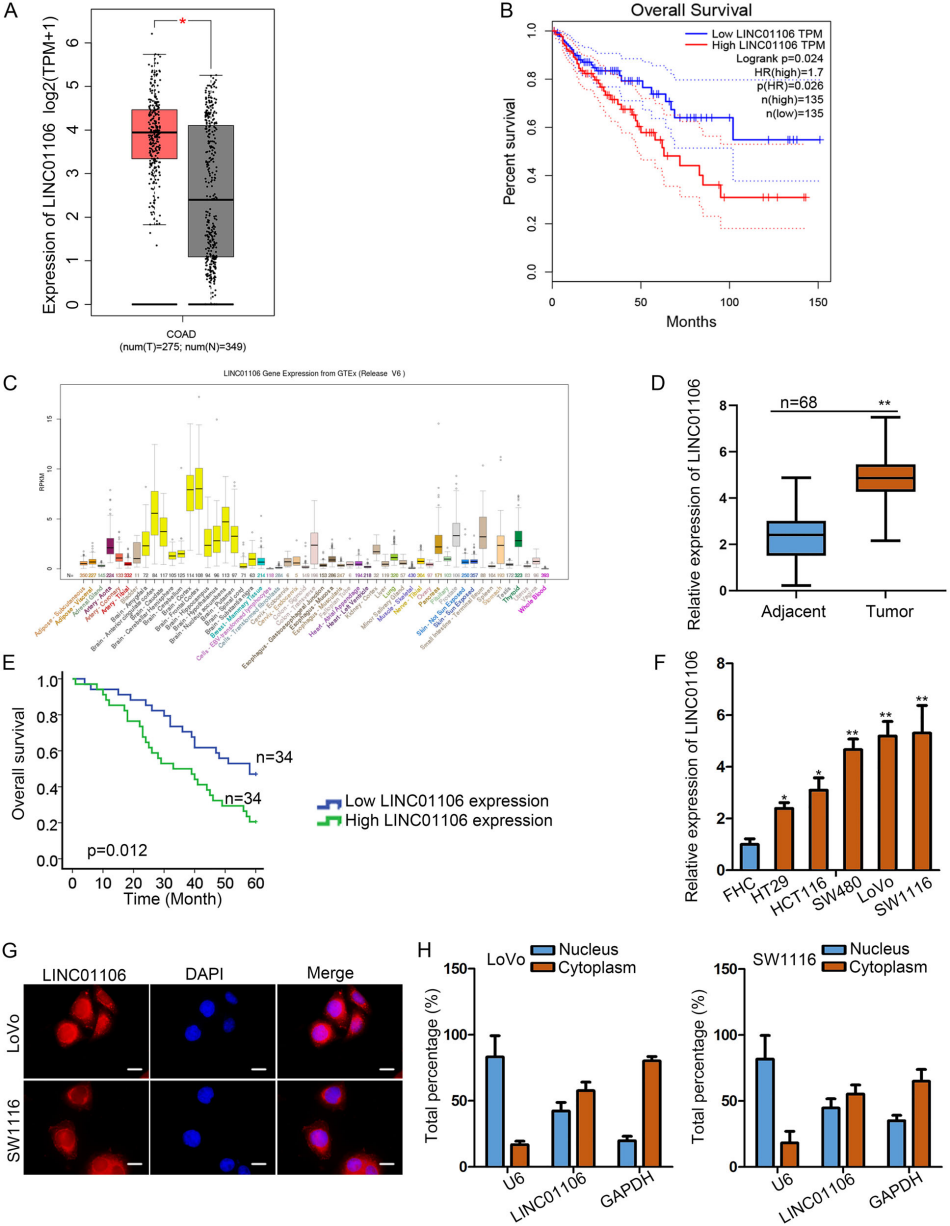
Highly expressed LINC01106 is correlated with the prognosis of CRC patients. **a** The expression profile of LINC01106 was observed in COAD samples and adjacent normal tissues from GEPIA. **b** Survival curve of COAD patients was generated based on the mean expression level of LINC01106 in patient samples of GEPIA. **c** LINC01106 presented the low expression in normal colon tissues based on the data of UCSC. **d** The expression of LINC01106 in 68 pairs of CRC tissues was analyzed via qRT-PCR. **e** Kaplan–Meier curve showed the clinical significance of LINC01106 in CRC patients. **f** LINC01106 expression level was examined in CRC cells (HT29, HCT116, SW480, LoVo, SW1116) using qRT-PCR by comparing with FHC cell (normal control). **g** and **h** Through FISH assay and subcellular distribution, the localization of LINC01106 is presented in both cytoplasm and nucleus. Data shown as mean ± SD were collected from three independent experiments. ^*^P < 0.05, ^**^P < 0.01 indicated data had statistical significance.

Through screening on online database GEPIA (http://gepia2.cancer-pku.cn/#analysis), LINC01106 was identified to be up-regulated in colon adenocarcinoma (COAD) samples compared to normal ones (Fig. 1a). Moreover, higher level of LINC01106 was correlated with worse prognosis of COAD patients (Fig. 1b). According to the data searched from UCSC (http://genome.ucsc.edu/), LINC01106 expression was lower in normal colon tissues (Fig. 1c). Meanwhile, we also determined the upregulation of LINC01106 in 68 CRC tissues compared to adjacent normal tissues (Fig. 1d). Consistently, high expression of LINC01106 was closely correlated with the poor prognosis in 68 CRC patients (Fig. 1e). Additionally, high level of LINC01106 in CRC cells (HT29, HCT116, SW480, LoVo, SW1116) were validated by qRT-PCR (Fig. 1f). Besides, the enhancement of LINC01106 expression in CRC cells relative to normal FHC cells was further proven by agarose gel eletrophoresis (Fig. S1A). Through FISH assay and subcellular fractionation assay, LINC01106 was identified to be distributed in both cytoplasm and nucleus (Fig. 1g, h), and this phenomenon was further verified by agarose gel eletrophoresis (Fig. S1B). These results prompted us to further investigate the association of LINC01106 with CRC progression.

### Depletion of LINC01106 attenuates the malignant phenotypes of CRC cells

**Fig. 2:**
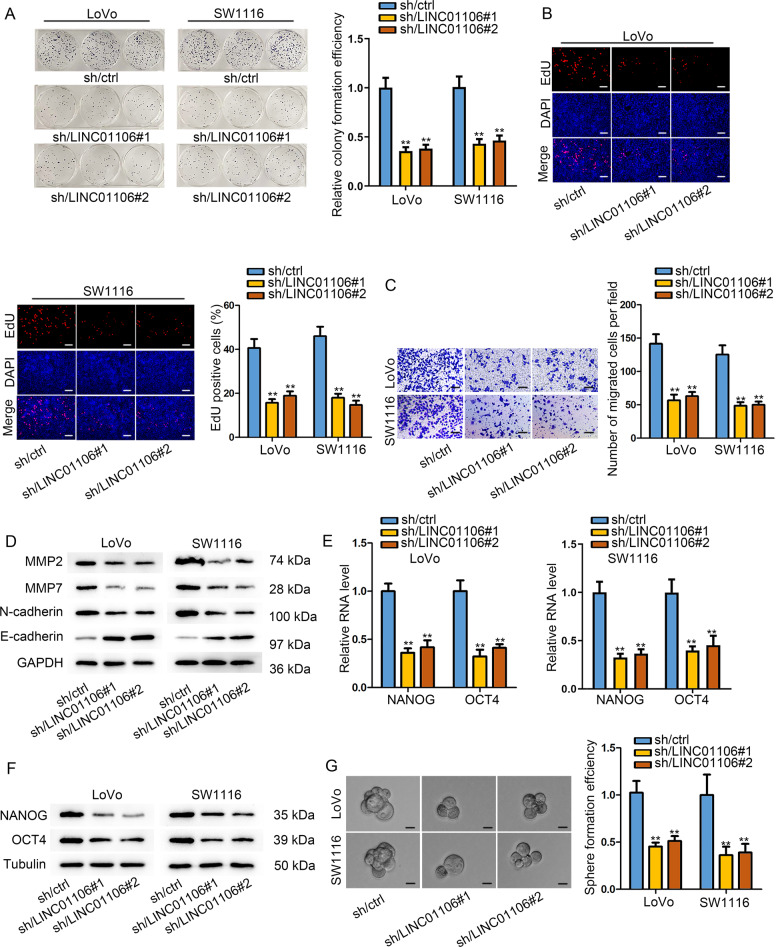
Depletion of LINC01106 attenuates the malignant phenotypes of CRC cells. **a** Colony-forming ability of LoVo and SW1116 cells was assessed after loss of LINC01106. **b** EdU assay revealed the proliferative ability of LoVo and SW1116 cells in response to silencing of LINC01106. **c** Transwell migration assay in LoVo and SW1116 cells transfected with sh-LINC01106 or sh-NC. **d** Western blot assay of the levels of migration-related proteins (MMP2 and MMP7) and EMT markers (E-cadherin and N-cadherin) in response to LINC01106 blockade. **e** and **f** The expression of stemness markers (NANOG and OCT4) was analyzed via qRT-PCR and western blot after LINC01106 depletion. **g** Sphere formation of LINC01106-silenced cells was analyzed compared with sh-NC group cells. Data shown as mean ± SD were collected from three independent experiments. ^**^P < 0.01 indicated data had statistical significance.

To identify the role of LINC01106 in CRC cells, we designed and executed loss-of function assays. At first, we used shRNAs against LINC01106 to efficiently deplete LINC01106 expression in LoVo and SW1116 cells (Fig. S1C). Loss of LINC01106 remarkably decreased the colony-forming ability of LoVo and SW1116 cells (Fig. 2a). Consistently, EdU assay demonstrated that silencing of LINC01106 obviously restricted cell proliferation (Fig. 2b). Transwell migration assay revealed that LoVo and SW1116 cells presented a lower potential to migrate after LINC01106 was down-regulated (Fig. 2c). Meanwhile, the levels of migration-related proteins (MMP2 and MMP7) were decreased in response to LINC01106 blockade. In addition, the increase of E-cadherin level and decrease of N-cadherin level were also observed in LINC01106-downreguated CRC cells, implying that EMT process was blocked by LINC01106 silencing (Fig. 2d). IF assay also elaborated that downregulation of LINC01106 led to the suppression on EMT process (Fig. S1D). Stem-like phenotype is an essential factor in promoting malignant progress of human cancers. The expression levels of stemness markers (NANOG and OCT4) were analyzed via qRT-PCR and western blot. Both levels of stemness markers were reduced, suggesting a restrained stem-like potential of LINC01106-silenced cells (Fig. 2e, f). As demonstrated in Fig. 2g, sphere formation of CRC cells was attenuated by the knockdown of LINC01106. These data suggested that knockdown of LINC01106 blocks CRC cell proliferation, migration, and stemness.

### Cytoplasmic LINC01106 has a positive effect on Gli4 expression by acting as a sponge for miR-449b-5p

**Fig. 3:**
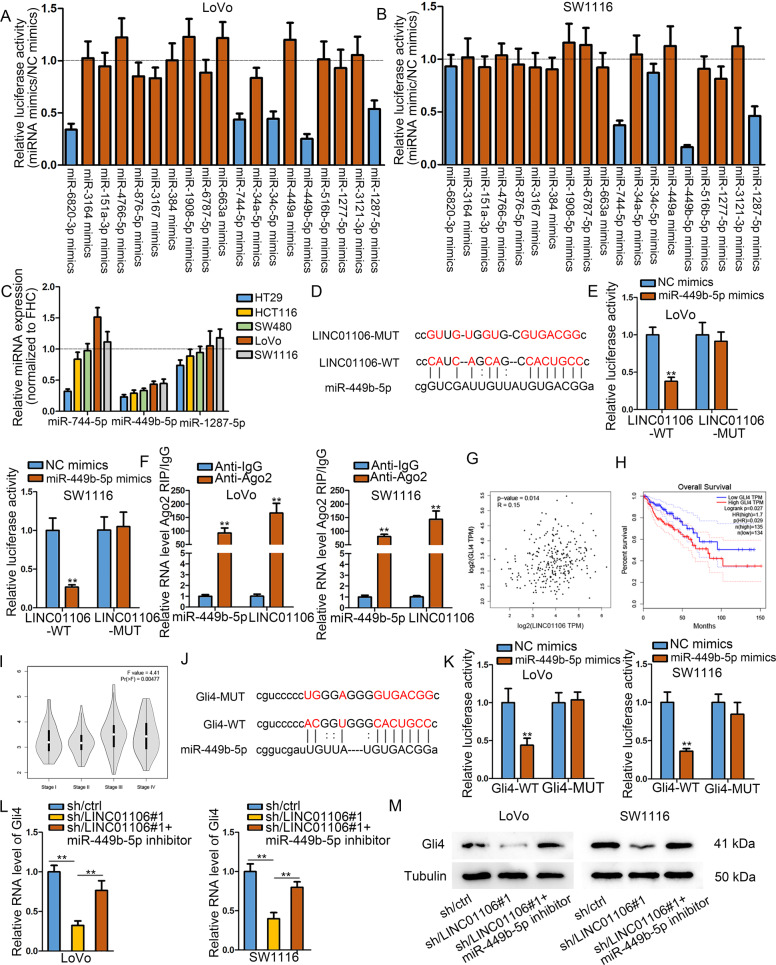
Cytoplasmic LINC01106 has a positive effect on Gli4 expression by acting as a sponge for miR-449b-5p. **a** and **b** Luciferase activity of vector containing the whole length of LINC01106 in both LoVo and SW1116 transfected with miRNA mimics specific to 10 candidate miRNAs. **c** Three miRNAs were examined by qRT-PCR in normal cell line and dive CRC cells. **d** The binding sites of LINC01106 to miR-449b-5p were predicted. **e** Luciferase reporter assay introduced that activity of LINC01106-WT reporter or LINC01106-MUT reporter vectors in response to miR-449b-5p overexpression. **f** RIP assay elucidated that both LINC01106 and miR-449b-5p were enriched in RNA-induced silencing complex (RISC). **g** A positive correlation between LINC01106 and Gli4 expression was revealed in COAD tissues using GEPIA. **h** GEPIA showed that Gli4 had a close association with low overall survival rate of COAD patients. **i** Highly expressed Gli4 was found by TCGA related to advanced stages in COAD patients. **j** The sites in Gli4 3’UTR responsible for binding miR-499b-5p were shown and mutation at this region was performed. **k** Subsequent luciferase reporter assay verified that miR-499b-5p mimics declined the luciferase activity of Gli4-WT, whereas mutation at this place canceled this impact. **l** and **m** The mRNA and protein levels of Gli4 were measured in CRC cells transfected with sh-NC, sh-LINC01106, or co-transfected with sh-LINC01106 and miR-449b-5p inhibitors. Data shown as mean ± SD were collected from three independent experiments. ^**^P < 0.01 indicated data had statistical significance.

After identification of LINC01106 function in CRC, we continued to explore its regulatory mechanism. Competing endogenous RNA (ceRNA) mechanism has been widely reported as a post-transcriptional regulatory mechanism. Based on this point, we explored the downstream miRNAs of LINC01106 from starBase (http://starbase.sysu.edu.cn/). Nineteen miRNAs with the LINC01106-binding potential were searched out and subjected to luciferase reporter assays. As depicted in Fig. 3a, b, overexpression of three miRNAs (miR-744-5p, miR-449b-5p, and miR-1287-5p) could reduce the luciferase activity of vector containing the whole length of LINC01106 in both LoVo and SW1116 cells. Next, the expression levels of these three candidate miRNAs were analyzed by qRT-PCR. It was uncovered that miR-449b-5p was significantly down-regulated in five CRC cells compared to FHC cells (Fig. 3c). Furthermore, we discovered miR-449b-5p was down-regulated in CRC tissues relative to adjacent normal ones and its low expression was associated with the poor survival in 68 CRC patients (Fig. S1E, F). Meanwhile, we observed that miR-449b-5p level was elevated due to LINC01106 depletion (Fig. S1G). Therefore, we selected miR-449b-5p as subsequent research object. The binding sequence of miR-449b-5p in LINC01106 was obtained and mutated for mechanism of investigation (Fig. 3d). After confirming the up-regulation of miR-449b-5p upon transfection with miR-449b-5p mimics (Fig. S1H), we performed luciferase reporter assays and results revealed that the luciferase activity of LINC01106-WT reporter was declined after miR-449b-5p overexpression (Fig. 3e). Ago2-RIP assay revealed that LINC01106 and miR-449b-5p were enriched in RNA-induced silencing complexes (RISCs) (Fig. 3f). Above data suggested the sponge role of LINC01106 for miR-449b-5p. Subsequently, we searched the target mRNAs of miR-449b-5p. Screening from three bioinformatics websites, there were 70 potential downstream targets of miR-449b-5p (Fig. S2A). Among these 70 candidate targets, Gli4 was positively correlated with LINC01106 in TCGA COAD samples and had a close association with low overall survival rate of COAD patients (Fig. 3g, h). Moreover, highly expressed Gli4 was found to be significantly related to advanced stages in TCGA COAD samples (Fig. 3i). Consistently, Gli4 was highly expressed in 68 CRC tissues and increased Gli4 level might predict a poor outcome in CRC patients (Fig. S2B, C). The binding sites of miR-499b-5p in Gli4 3′UTR were shown (Fig. 3j). Subsequent luciferase reporter assay verified that miR-499b-5p mimics led to the declined luciferase activity of Gli4-WT reporter (Fig. 3k). The down-regulation of miR-449b-5p by specific miRNA inhibitor was identified with qRT-PCR (Fig. S2D). Next, we detected the influence of miR-499b-5p on Gli4 expression. With the up-regulation of miR-499b-5p, Gli4 expression was repressed at mRNA and protein levels (Fig. S2E). Furthermore, the luciferase activity of Gli4-WT reporter decreased by miR-449b-5p mimics was recovered after co-transfection with pcDNA3.1-LINC01106 (Fig. S2F). Finally, depletion of LINC01106 led to the reduction of Gli4 mRNA and protein, whereas suppression of miR-499b-5p could relieve this reduction (Fig. 3l, m). According to these findings, we summarized that LINC01106 enhances Gli4 level by sponging miR-449b-5p.

### Elevation of Gli4 reverses the LINC01106 knockdown-induced progression retard in CRC

**Fig. 4:**
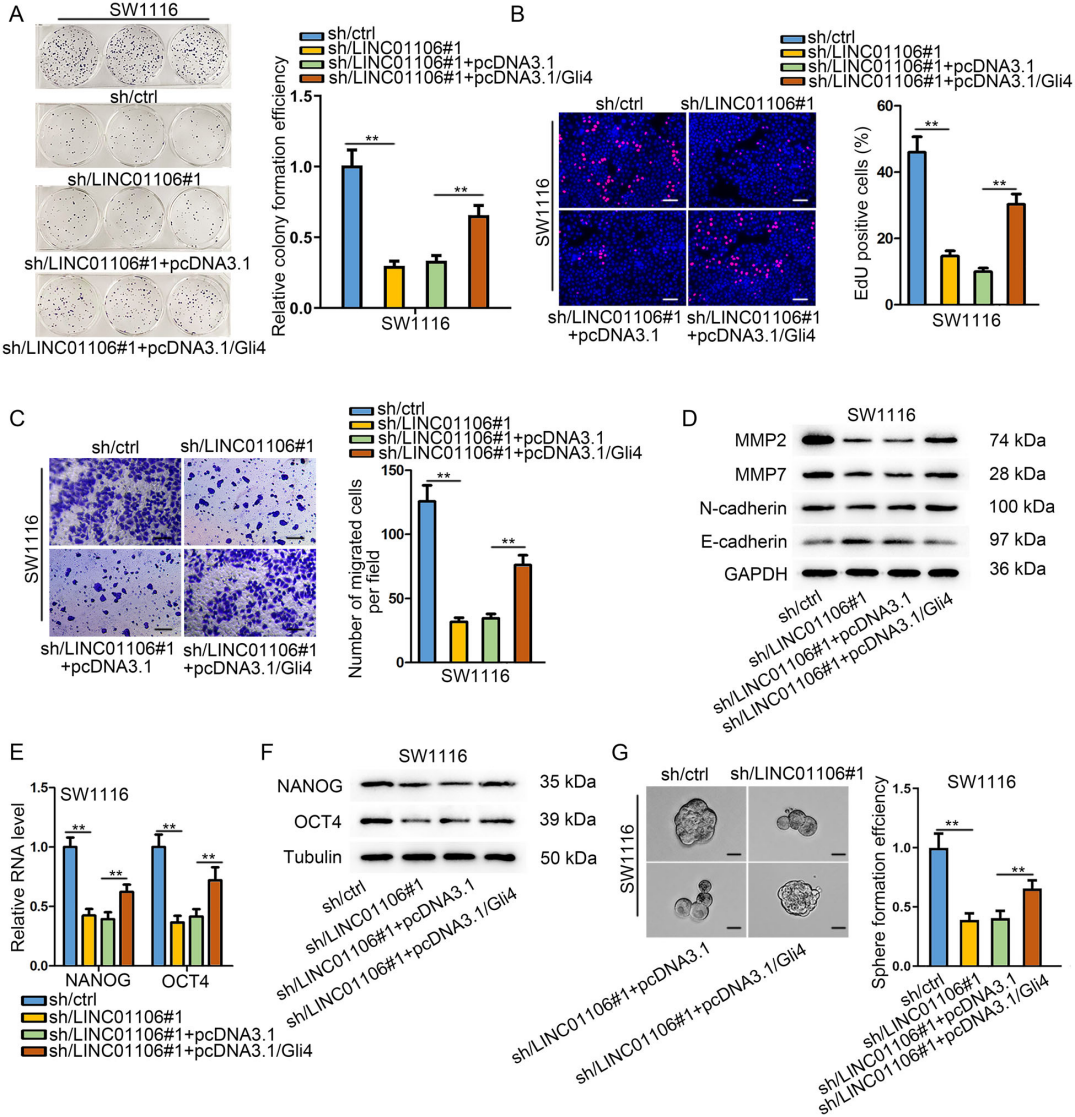
Elevation of Gli4 reverses the LINC01106 knockdown-induced progression retard in CRC. **a** and **b** As it turned out in colony formation and EdU assays, LINC01106 silencing-caused proliferation suppression could be reversed by overexpressing Gli4. **c** In Transwell migration assay, suppressed CRC cell migration resulted from LINC01106 blockade was promoted via pcDNA3.1/Gli4. **d** Western blot assay was used to examine migrating-related proteins and EMT markers in cells transfected with sh-LINC01106 or co-transfected with sh-LINC01106 and pcDNA3.1/Gli4. **e** and **f** Analyses by qRT-PCR and western blot indicated depressed level of stemness-related factors in LINC01106-knockdown cells was restored via pcDNA3.1/Gli4. **g** Sphere formation assay showed that pcDNA3.1/Gli4 attenuated the effects of LINC01106 knockdown on CRC cell sphere formation. Data shown as mean ± SD were collected from three independent experiments. ^**^P < 0.01 indicated data had statistical significance.

Functional assays were applied to validate that LINC01106 could regulate Gli4 in CRC cell growth. Firstly, we overexpressed Gli4 in CRC cells (Fig. S2G). It turned out that overexpression of Gli4 partly reversed the effect of LINC01106 silencing on proliferation suppression (Fig. 4a, b). Moreover, suppression on CRC cell migration resulted from LINC01106 blockade was partially attenuated via upregulation of Gli4 (Fig. 4c). Western blot assay uncovered that the inhibitory effect of LINC01106 deficiency on migrating-related proteins was abolished via the ectopic expression of Gli4. Furthermore, silenced LINC01106-induced EMT suppression could be antagonized by pcDNA3.1/Gli4 (Fig. 4d). We also disclosed that depressed expression of stemness markers in LINC01106-downregulated cells was restored via overexpression of Gli4 (Fig. 4e, f). Consistently, we also proved that upregulation of Gli4 attenuated the suppressive effects of LINC01106 knockdown on sphere formation efficiency (Fig. 4g). Together, cytoplasmic LINC01106 facilitates CRC progression through augmenting Gli4 expression.

### Nuclear LINC01106 recruits FUS to transcriptionally activate Gli1 and Gli2

**Fig. 5:**
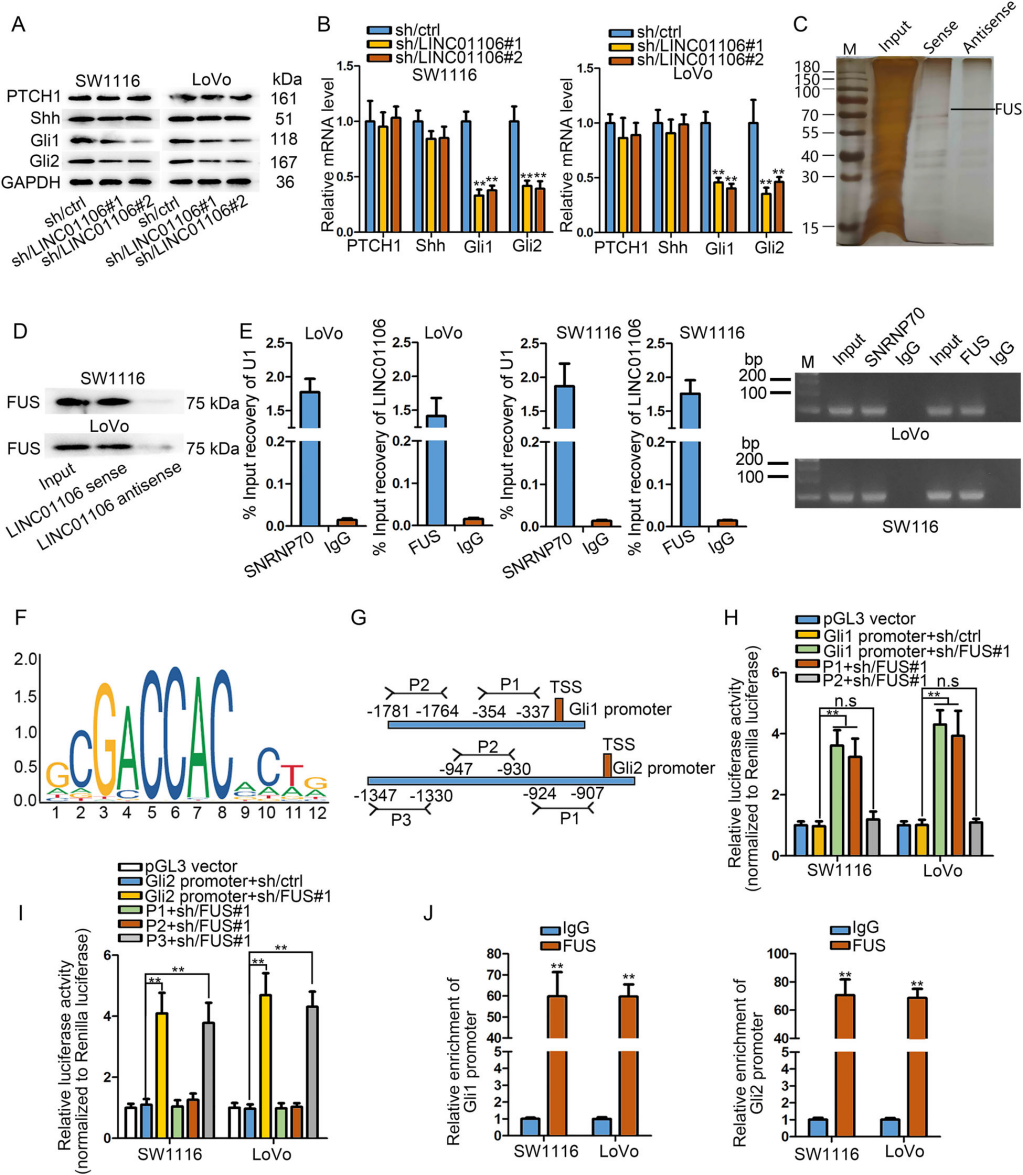
Nuclear LINC01106 recruits FUS to transcriptionally activate Gli1 and Gli2. **a** Western blot assay identified that LINC01106 suppression affected the level of Gli1 and Gli2 protein, but not PTCH1 and Shh. **b** The effect of LINC01106 on the mRNA level of Hedgehog pathway factors was detected by qRT-PCR. **c** RNA pull-down followed by silver staining exhibited the LINC01106-interacting proteins. **d** and **e** RNA pull-down followed by western blot assay and RIP assay together supported that LINC01106 combined with FUS. **f** Using JASPAR online tools, the DNA motif of FUS was presented. **g** The FUS-binding sites in Gli1 and Gli2 promoters were displayed. **h** and **i** The specific functional binding region in Gli1 and Gli2 promoters was confirmed by conducting luciferase reporter assays. **j** ChIP assay described the occupancy of FUS in the promoter region of Gli1 and Gli2. Data shown as mean ± SD were collected from three independent experiments. ^**^P < 0.01 indicated data had statistical significance. n.s. indicated difference had no statistical significance.

According to the results indicated in Fig. 4, Gli4 only could partially rescue the functions attenuated by LINC01106 depletion. Thus, we further detected whether other molecular mechanism involved in LINC01106-mediated CRC progression. GLI families are crucial downstream factors of hedgehog pathway which has been implicated in CRC^28^. Hence, we explored the association of LINC01106 with Hedgehog pathway. Western blot assay identified that LINC01106 suppression resulted in the decline of Gli1 and Gli2 proteins, while no impacts were observed on PTCH1 and Shh expressions (Fig. 5a). Furthermore, we detected the effect of LINC01106 on the mRNA levels of Hedgehog pathway factors. Silenced LINC01106 induced the down-regulation of Gli1 and Gli2, but had no regulatory effects on PTCH1 and Shh (Fig. 5b). Thus, Gli1 and Gli2 were supposed to be two potential downstream targets of LINC01106. At first, LINC01106 was identified as a positive regulator on the activity of Gli1 and Gl2 promoters (Fig. S3A). RNA pull-down followed by silver staining exhibited the LINC01106-interacting proteins (Fig. 5c). After mass-spectrometry analysis, three interacted proteins, including FUS, TAF15, and TCF4, were searched out due to their transcriptional regulation potential. To analyze whether they could regulate Gli1/2, we silenced these three factors with indicated shRNAs (Fig. S3B). As a result, only FUS silencing could efficiently reduce the expression levels of both Gli1 and Gli2 (Fig. S3C, D). Thus, we chose FUS protein for further mechanism investigation. RNA pull-down assay and RIP assay supported the interaction between LINC01106 and FUS (Fig. 5d, e). Then, we further detected the regulatory pattern of FUS on Gli1/2. As the results shown in Fig. S3E, FUS positively regulated the transcriptional activity of both Gli1 and Gli2. Since FUS has been reported as a RNA-binding protein that can bind to mRNA 3′UTR, we also applied luciferase reporter assay to detect whether FUS could affect the activity of Gli1 or Gli2 3′UTR. Intriguingly, FUS had no significant effect on the activity of Gli1 or Gli2 3′UTR, excluding the regulation of FUS on their stability (Fig. S3F). We also examined FUS expression in response to the knockdown of LINC01106. As a result, LINC01106 silencing had no significant effect on FUS expression (Fig. S3G). Thus, we speculated that LINC01106 recruited FUS to Gli1/2 promoters. The DNA motif of FUS was presented using JASPAR online tool (Fig. 5f). The binding sites of FUS in Gli1 and Gli2 promoters were also displayed (Fig. 5g). After successful overexpression of FUS (Fig. S3H), we carried out luciferase reporter assays. It suggested that overexpression of FUS increased the luciferase activity of reporter vector containing the whole Gli1 promoter or P1 region, indicating that P1 region was responsible for the interaction between FUS and Gli1 promoter (Fig. 5h). Meanwhile, we also determined that P3 of Gli2 promoter was the actual binding region for FUS (Fig. 5i). ChIP assay also validated the occupancy of FUS in the promoter region of Gli1 and Gli2 (Fig. 5j). Finally, we also explored whether Gli1 and Gli2 could be targeted by miR-449b-5p. As a result, the luciferase activity of reporters containing Gli1 or Gli2 3′UTR was not changed after miR-449b-5p was upregulated (Fig. S3I), excluding the post-transcriptional regulatory effect of miR-449b-5p on Gli1 and Gli2. These findings showed that LINC01106 transcriptionally activates Gli1 and Gli2 via recruiting FUS.

### GLi1/2/4 are three downstream factors involved in LINC01106-mediated CRC functions

**Fig. 6:**
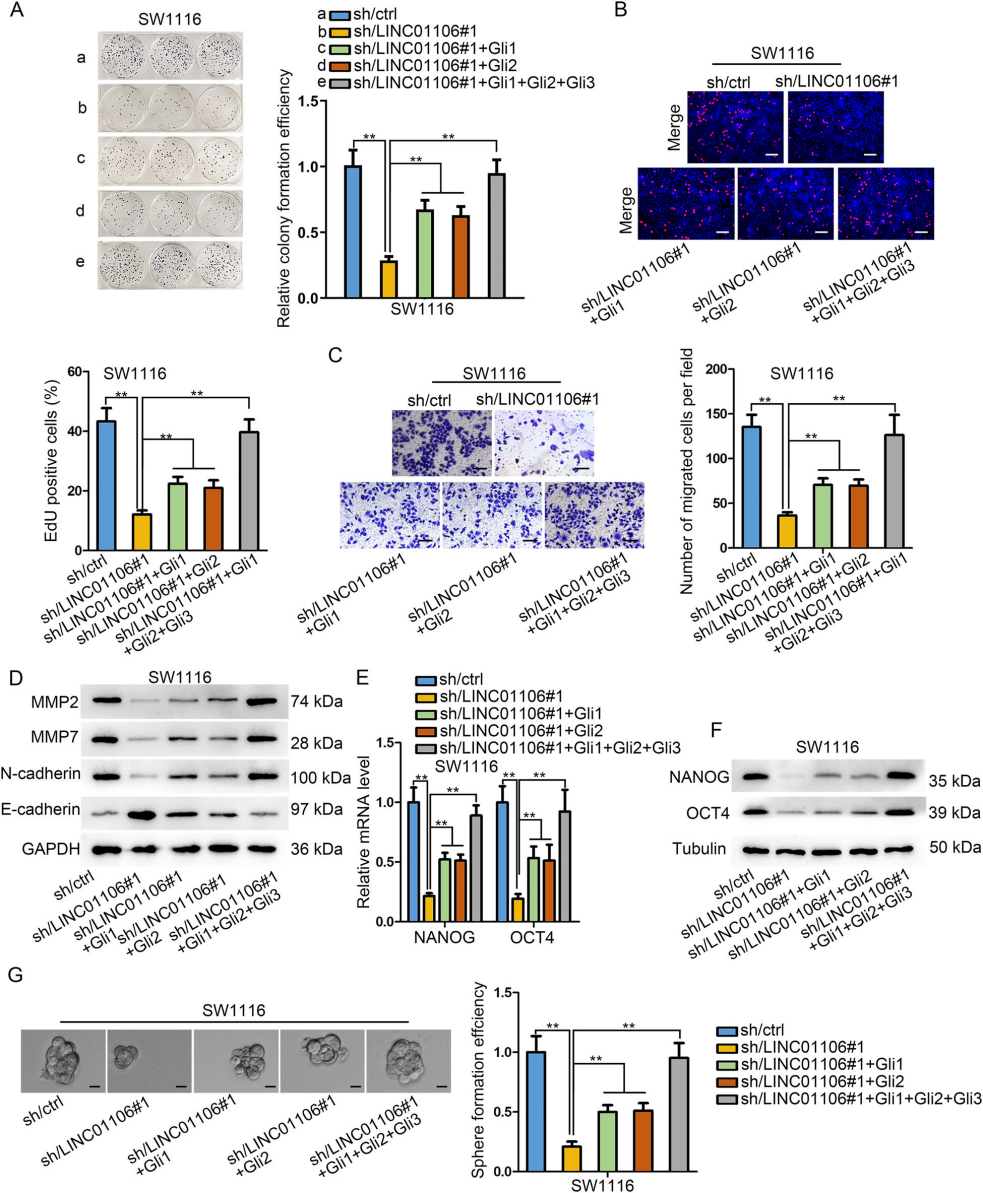
GLi1/2/4 are three downstream factors involved in LINC01106-mediated CRC functions. **a** and **b** Colony formation and EdU assays proved that Gli1, Gli2 partially rescued the suppressed proliferation in LINC01106-deficient SW1116 cells, whereas cell proliferation was totally rescued after overexpression of Gli1/2/4. **c** and **d** Transwell migration assay and western blot analysis validated that the introduction of pcDNA3.1/Gli1, pcDNA3.1/Gli2 partly recovered cell migration and EMT process of LINC01106-deficient SW1116 cells, but total recovered effect was observed when overexpressing Gli1/2/4. **e**–**g** Stemness of CRC cells suppressed by sh-LINC01106 was partly recovered by overexpression of Gli1 or Gli2 alone, but entirely rescued by overexpression of Gli1/2/4. Data shown as mean ± SD were collected from three independent experiments. ^**^P < 0.01 indicated data had statistical significance.

Rescue assays were carried out to testify the involvement of Gli1 and Gli2 in LINC01106-mediated CRC progression. Gli1 and Gli2 were overexpressed in CRC cells before rescue assays (Fig. S4A). Colony formation assay and EdU assay proved that Gli1, Gli2 partially rescued the suppressed proliferation in LINC01106-deficient SW1116 cells, whereas cell proliferation was totally rescued after co-overexpression of Gli1/2/4 (Fig. 6a, b). Transwell migration assay and western blot analysis validated that the introduction of pcDNA3.1/Gli1, pcDNA3.1/Gli2 partly recovered cell migration and EMT process of LINC01106-deficient SW1116 cells, but total recovered effect was observed when simultaneously overexpressing Gli1/2/4 (Fig. 6c, d). Meanwhile, stemness of CRC cells attenuated by silenced LINC01106 was partly recovered by the overexpression of Gli1 or Gli2 alone, but entirely rescued by the co-overexpression of Gli1/2/4 (Fig. 6e–g). Our observations indicated that LINC01106 regulates CRC progression through upregulating Gli1, Gli2, and Gli4.

### Gli2 transcriptionally activates LINC01106

**Fig. 7:**
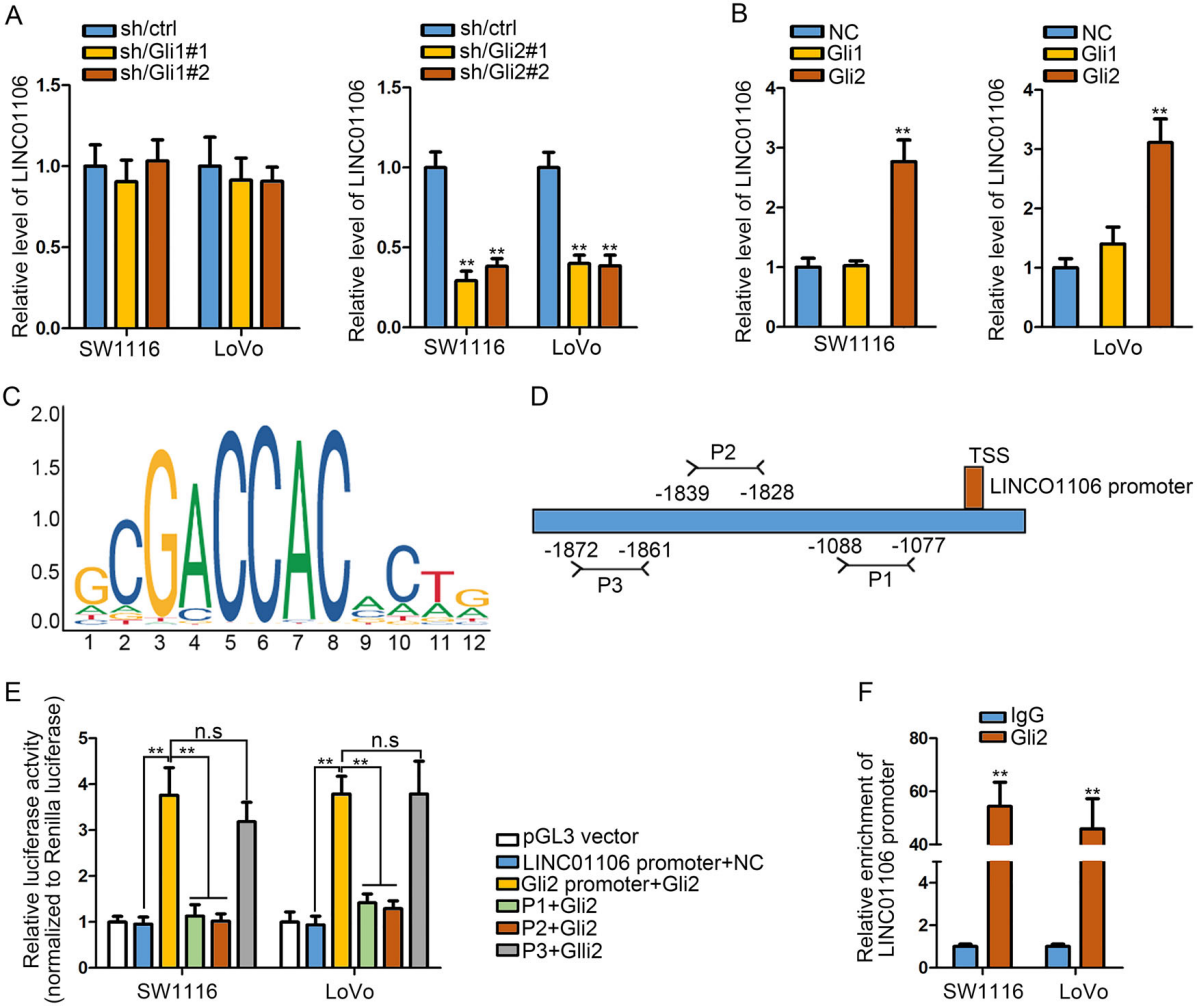
Gli2 transcriptionally activates LINC01106. **a** Result of qRT-PCR indicated LINC01106 expression in cells with or without the silencing of Gli1/2. **b** Augmentation of Gli2 elevated LINC01106 expression, as assessed by qRT-PCR. **c** Gli2 DNA motif was shown. **d** Potential binding regions in LINC01106 promoter for Gli2 (P1–P3) were exhibited. **e** Luciferase reporter assay demonstrated the functional region of Gli2 in LINC01106 promoter. **f** ChIP assay supported the Gli2 enrichment in LINC01106 promoter. Data shown as mean ± SD were collected from three independent experiments. ^**^P < 0.01 indicated data had statistical significance. n.s. indicated difference had no statistical significance.

**Fig. 8:**
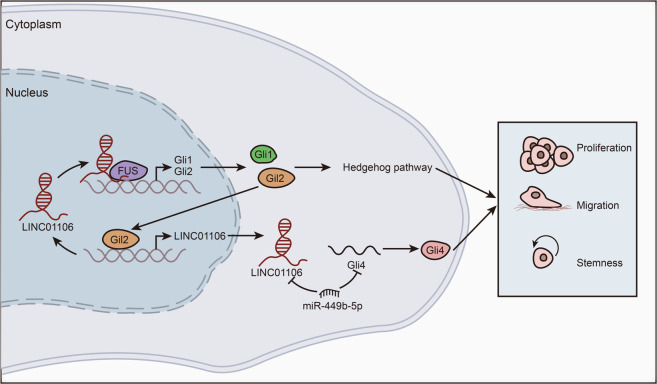
A schematic diagram illustrated the mechanism by which LINC01106 promoted CRC progression. Graphical abstract illustrated that Gli2-induced activation of LINC01106 promoted CRC progress via regulating Gli1/2/4.

Gli1 and Gli2 have been reported as two transcriptional factors^29^. Here, we speculated whether Gli1 and Gli2 could regulate the transcription of LINC01106. Gli1 and Gli2 were separately silenced in two CRC cells for further analysis (Fig. S4B). We detected the level of LINC01106 in cells with the silencing or overexpression of Gli1/2. Consequently, LINC01106 level was obviously restrained by Gli2 silencing but was strengthened by Gli2 overexpression (Fig. 7a, b). Gli2 DNA motif was obtained and shown in Fig. 7c. Potential binding regions in LINC01106 promoter for Gli2 (P1–P3) were also exhibited (Fig. 7d). Luciferase reporter assay unveiled that silencing Gli2 led to the suppression on the activity of LINC01106 promoter and also the P3 of LINC01106 promoter. However, overexpression of Gli2 led to the opposite results (Fig. 7e). Moreover, results obtained from ChIP assay further confirmed the affinity of Gli2 to LINC01106 promoter (Fig. 7f). Our data suggested that Gli2-induced the transcriptional activation of LINC01106 promotes CRC progression via regulating Gli1/2/4 (Fig. 8).

## Discussion

Studies on lncRNAs have elaborated that lncRNAs involve in CRC onset and aggravation. As previously proven, lncRNA CASC19 is an oncogene in CRC through miR-140-5p/CEMIP axis^30^ or via interacting with CPSF3^31^. LINC01354 activates Wnt/β-catenin signaling via binding to hnRNP-D and facilitates CRC growth and aggressiveness^32^. To our knowledge, LINC01106 is a novel lncRNA that has not been clarified in human cancers. In our current study, we found that LINC01106 was differentially expressed in TCGA COAD samples and predicted poor prognosis in COAD patients with the aid of online public tools. We also provided clinical data to support the results of online prediction. Similarly, high level of LINC01106 was also examined in the clinical CRC samples obtained from 68 patients. Functionally, differentially expressed lncRNAs are known as oncogenic factors in various human cancers^19,33–35^ In this study, we determined that LINC01106 exerted oncogenic role in CRC through promoting cell proliferation, mobility, EMT, and stemness. Our study is the first one to reveal the functions of LINC01106 in CRC progression.

Currently, studies have elucidated that lncRNAs can act as sponges of miRNAs to enhance the expression of mRNA in tumor development^36^. In the current study, we found that LINC01106 could act as a miR-449b-5p sponge to upregulate Gli4 in CRC cells. Mechanically, we first unveiled the interaction between LINC01106 and miR-449b-5p. Importantly, we confirmed that LINC01106 exerts oncogenic functions in CRC partially through Gli4.

Activated Hedgehog pathway is pro-proliferative and stemness-facilitating in malignant tumors^37–39^. Given that LINC01106 positively regulated Gli4, we probed whether LINC01106 was correlated with Hedgehog pathway in CRC. We found that the expression level of PICTH1 and Shh was not changed, whereas the protein levels of Gli1 and Gli2 were significantly regulated by LINC01106. Furthermore, we determined that LINC01106 positively regulated the mRNA levels of both Gli1 and Gli2. Thus, we further investigated the regulatory mechanism by which LINC01106 positively regulated Gli1 and Gli2. It has been widely reported that lncRNAs can recruit specific proteins to manipulate the expression of their target genes^40,41^. For example, lncRNA GClnc1 is exemplified as a scaffold for WDR5 and KAT2A, thus promoting SOD2 expression in gastric carcinogenesis^16^. LncRNA AGAP2-AS1 recruits EZH2 and LSD1 to LATS2 and KLF2 promoters and thus represses their expressions in non-small-cell lung cancer^42^. Here, we observed that LINC01106 recruited FUS to Gli1 and Gli2 promoters for transcriptional activation of Gli1/2. It has been revealed by diverse reports, FUS can bind to gene promoter to affect gene transcription^43–46^. In our current study, we determined that LINC01106 recruited FUS to Gli1 and Gli2 promoters, thereby strengthening the expression of Gli1 and Gli2. Finally, we also proved the partial reversing effect of Gli1 and Gli2 on LINC01106-mediated CRC cell functions^47^. More importantly, co-overexpression of Gli1, Gli2, and Gli4 totally reversed the effect of silenced LINC01106 on CRC progression. Thus, we summarized that LINC01106 promotes CRC progression via inducing the upregulation of Gli1/2/4.

Upregulation of lncRNAs may be attributed to their upstream transcriptional activators, such as MYC-induced lncRNA SNHG15^48^ and FOXM1-activated LINC-ROR^49^. In the present study, we confirmed that Gli2 could facilitate the transcription of LINC01106. This is the first time to elucidate Gli2/LINC01106 axis in CRC.

In conclusion, we revealed a novel positive feedback loop of LINC01106/Gli2 and a post-transcriptional regulatory pathway in facilitating CRC growth and aggressiveness. Our study may contribute to providing more effective therapeutic targets for CRC. Nevertheless, lack of animal study is a limitation of the current study. We will establish animal model to demonstrate the effect of LINC01106 on in vivo tumor growth and metastasis.

## Supplementary information

Supplementary Figure 1

Supplementary Figure 2

Supplementary Figure 3

Supplementary Figure 4

Supplementary Figure legends
