# Supplementary figures and images for: LINC01106 drives colorectal cancer growth and stemness through a positive feedback loop to regulate the Gli family factors
Source: Cell Death Dis. 2020 Oct 16;11(10):869. doi: 10.1038/s41419-020-03026-3 (PMC7567881; doi:10.1038/s41419-020-03026-3)

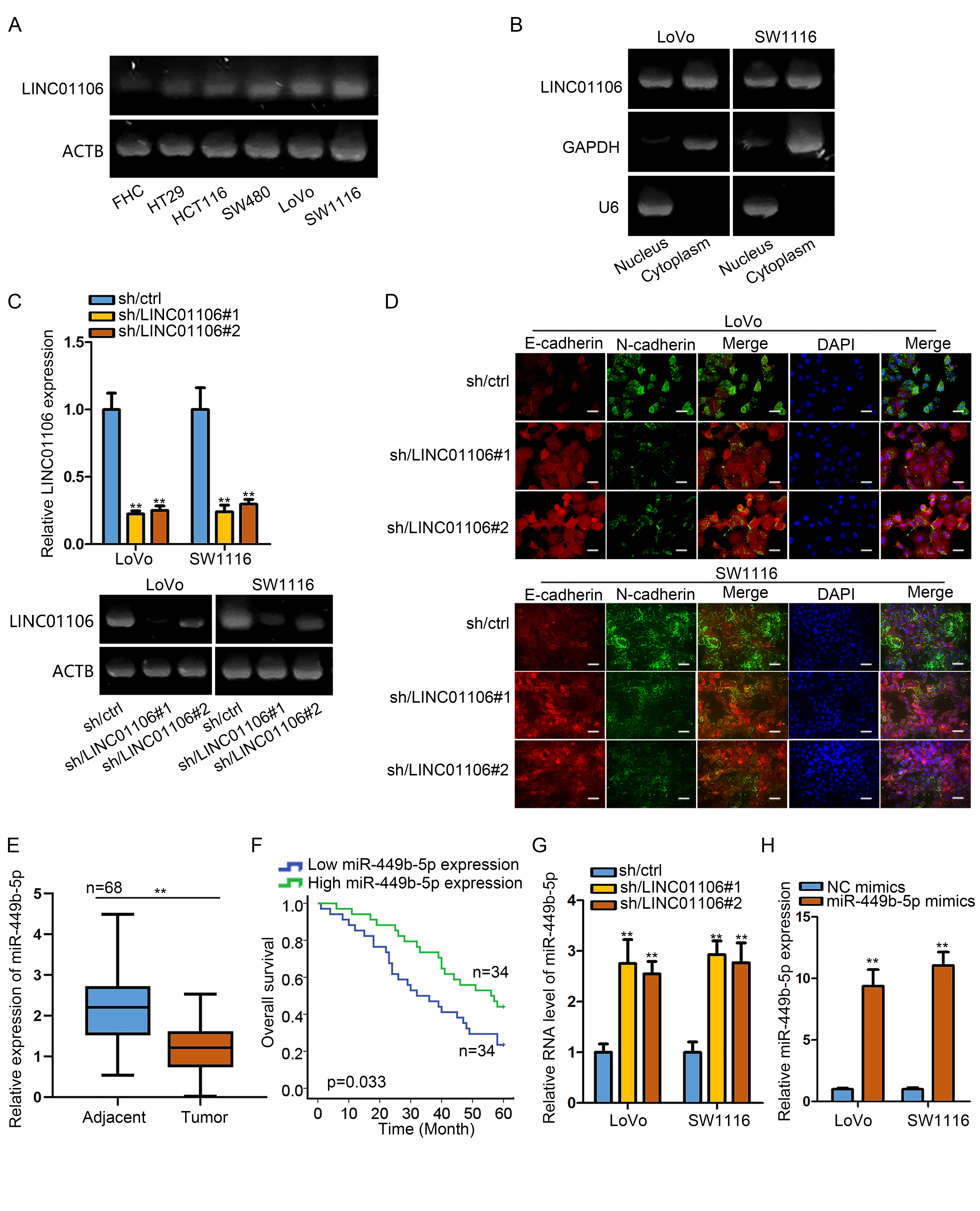

Supplement: Supplementary file 1 — Supplementary Figure 1 [file 41419_2020_3026_MOESM1_ESM.tif]

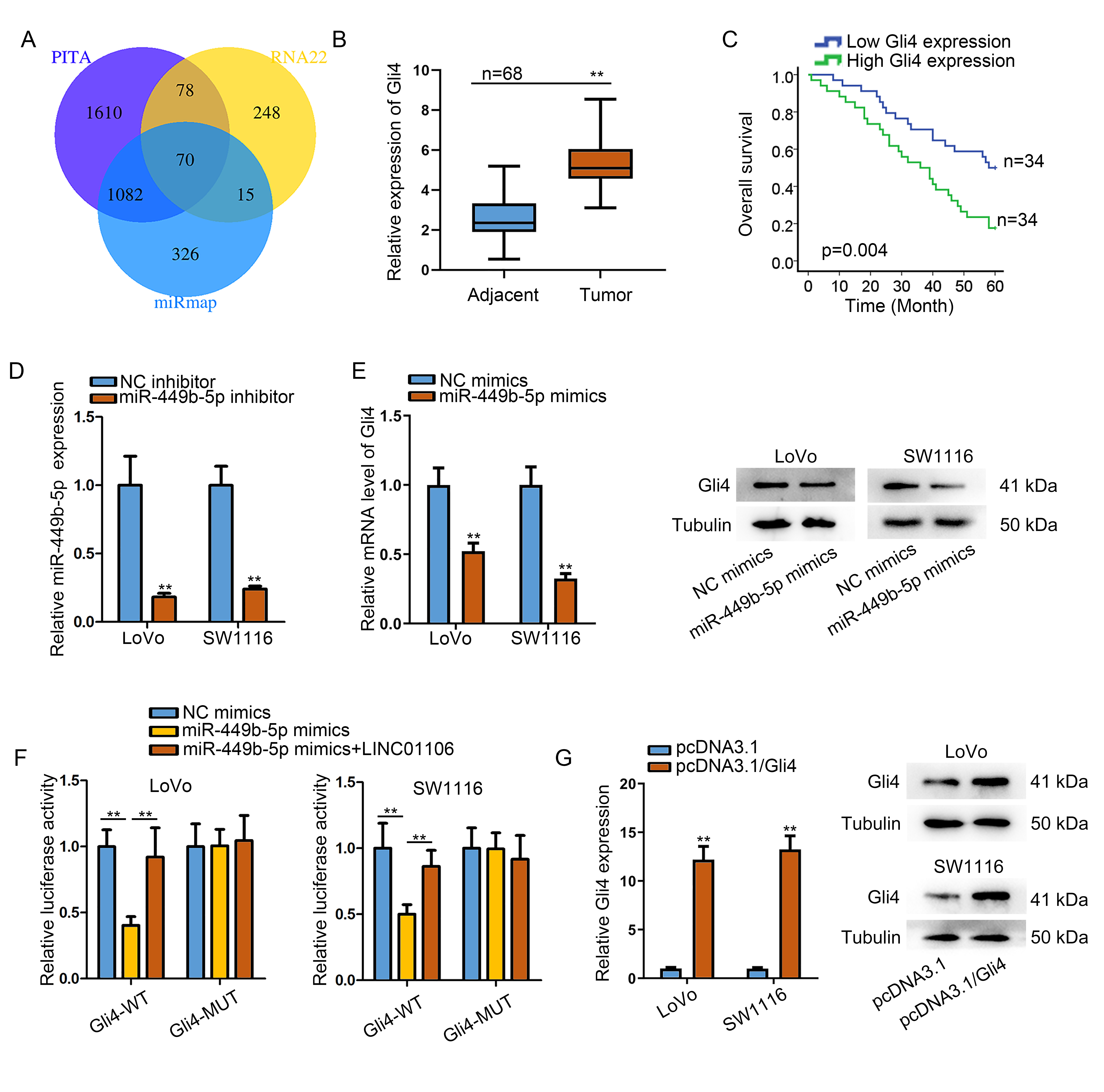

Supplement: Supplementary file 2 — Supplementary Figure 2 [file 41419_2020_3026_MOESM2_ESM.tif]

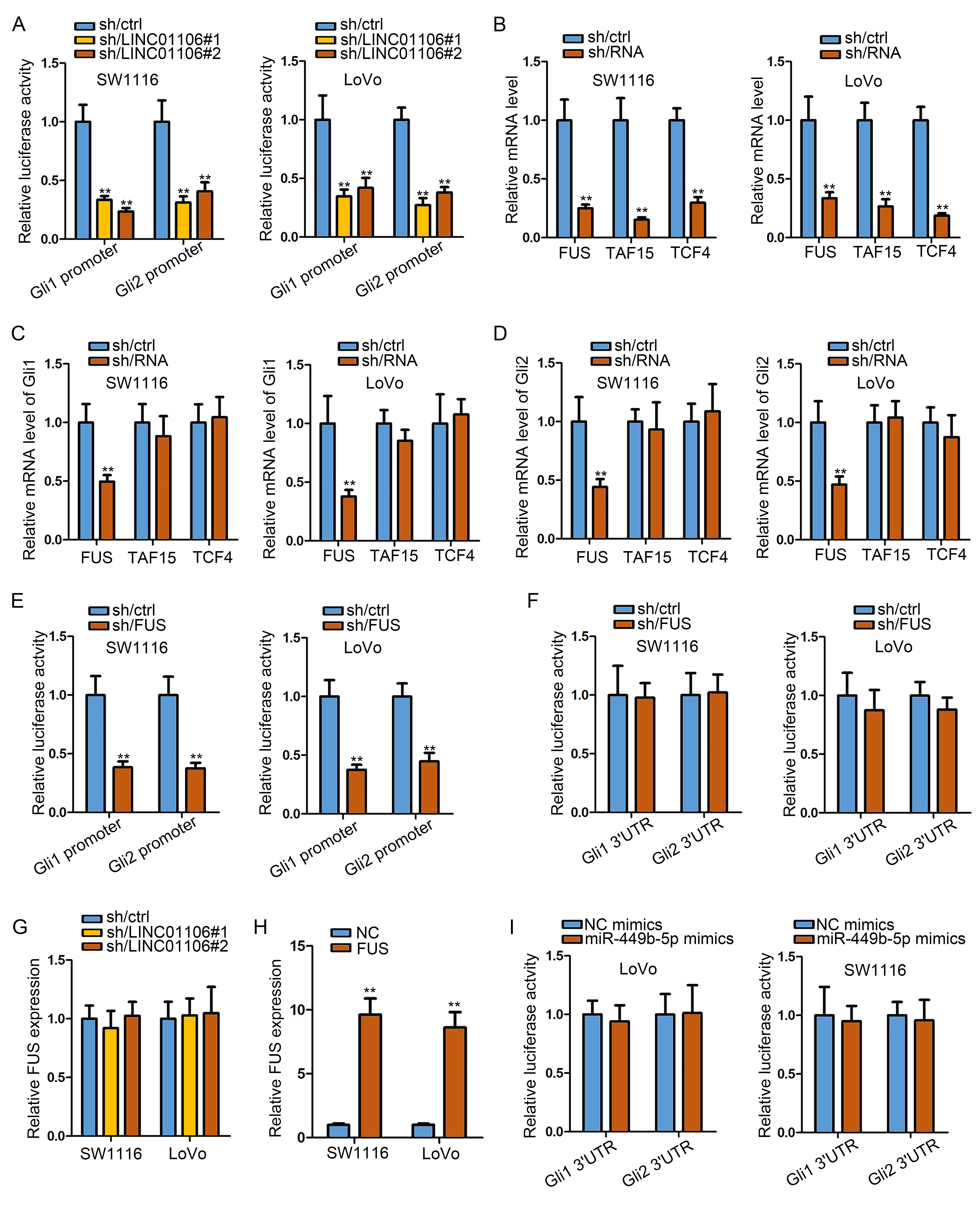

Supplement: Supplementary file 3 — Supplementary Figure 3 [file 41419_2020_3026_MOESM3_ESM.tif]

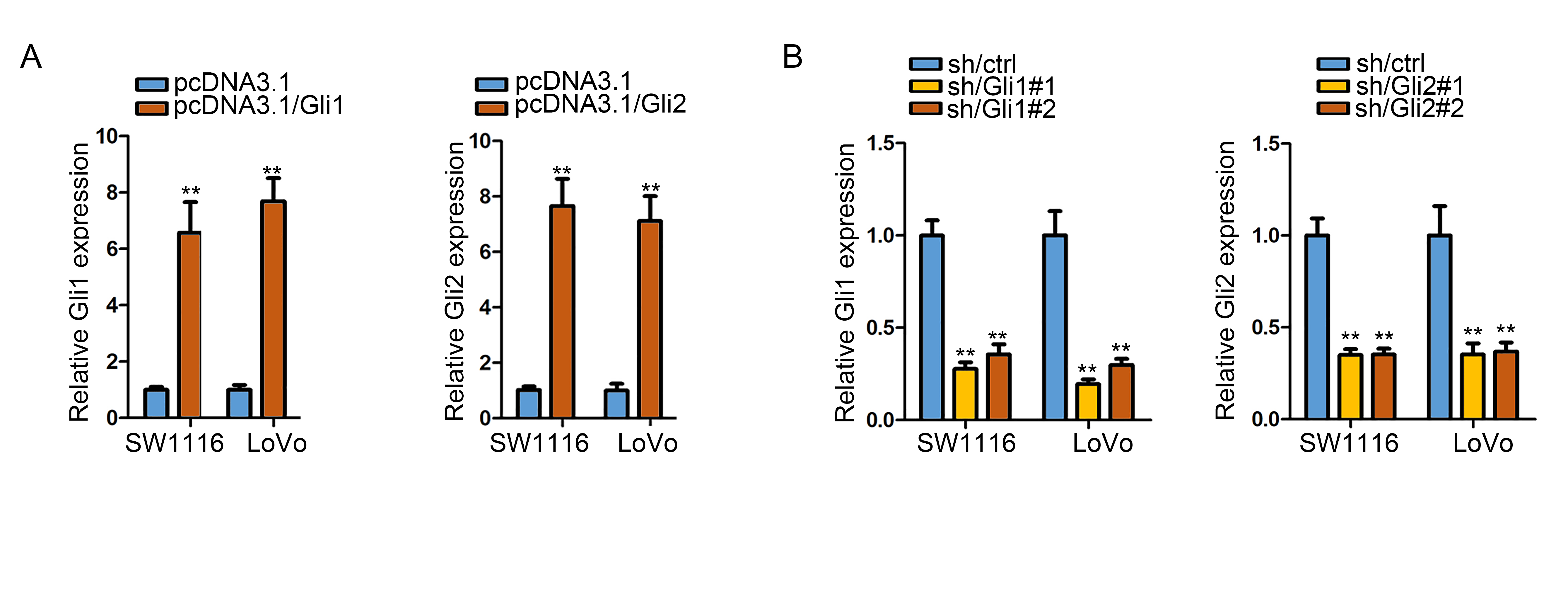

Supplement: Supplementary file 4 — Supplementary Figure 4 [file 41419_2020_3026_MOESM4_ESM.tif]
