# Supplementary material for: LINC01106 drives colorectal cancer growth and stemness through a positive feedback loop to regulate the Gli family factors
Source: Cell Death Dis. 2020 Oct 16;11(10):869. doi: 10.1038/s41419-020-03026-3 (PMC7567881; doi:10.1038/s41419-020-03026-3)
Supplement: Supplementary file 5 — Supplementary Figure legends [file 41419_2020_3026_MOESM5_ESM.docx]

**Figure S1**

(A) Agarose gel electrophoresis analysis of LINC01106 expression in CRC cell lines. (**B**) The level of LINC01106 in nucleus and cytoplasm of LoVo and SW1116 cells was detected via agarose gel electrophoresis. (**C**) Efficient depletion of LINC01106 in LoVo and SW1116 cells was confirmed by qRT-PCR and agarose gel electrophoresis. (**D**) Immunofluorescence assay in LINC01106-downregulated CRC cells to show the expression of EMT markers. (**E-F**) The expression of LINC01106 in 68 pairs of CRC tissues was assessed by qRT-PCR and the clinical significance of LINC01106 in CRC patients analyzed by Kaplan-Meier curve. (**G**) The impact of LINC01106 silencing on miR-449b-5p expression was probed by qRT-PCR. (**H**) The overexpression of miR-499b-5p was confirmed by using qRT-PCR analysis. Data shown as mean ± SD were collected from three independent experiments. ^**^P < 0.01 indicated data had statistical significance.

**Figure S2**

(**A**) 70 potentials downstream target of miR-449b-5p was screened out from three bioinformatics websites. (**B-C**) Gli4 expression and significance in clinical samples were respectively assessed by qRT-PCR and Kaplan-Meier analyses. (**D**) Inhibition efficiency of miR-449b-5p inhibitor was validated by qRT-PCR. (**E**) The effect of miR-449b-5p on Gli4 expression was examined by qRT-PCR and western blot. (**F**) Luciferase reporter assay validated the relationship among miR-449b-5p, LINC01106 and Gli4. (**G**) Overexpression efficiency of Gli4 confirmed by qRT-PCR and western blot. Data shown as mean ± SD were collected from three independent experiments. ^**^P < 0.01 indicated data had statistical significance.

**Figure S3**

(**A**) The effect of LINC01106 on the activity of Gli1 and Gl2 promoters was found to be positive. (**B**) Three potential TFs were silenced with indicated shRNAs. (**C-D**) The effect of three silenced TFs on the levels of Gli1 and Gli2. (**E**) The regulatory role of FUS on the activity of Gli1/2 promoter. (**F**) Luciferase reporter assay was applied to detect the potential effect of FUS on the activity of Gli1 or Gli2 3’UTR. (**G**) The expression of FUS in response to the knockdown of LINC01106. (**H**) Overexpression of FUS was successfully conducted. (**I**) Luciferase reporter assay presented that the luciferase activity of reporters containing Gli1 or Gli2 3’UTR was not changed after miR-449b-5p was up-regulated. Data shown as mean ± SD were collected from three independent experiments. ^**^P < 0.01 indicated data had statistical significance.

**Figure S4**

(**A**) Results of qRT-PCR indicated that Gli1 and Gli2 were overexpressed in LoVo and SW1116 cells. (**B**) Decreased levels of Gli1 and Gli2 in above two CRC cells were confirmed by qRT-PCR. Data shown as mean ± SD were collected from three independent experiments. ^**^P < 0.01 indicated data had statistical significance.
